# Clinical, demographic and laboratory parameters at HAART initiation associated with decreased post-HAART survival in a U.S. military prospective HIV cohort

**DOI:** 10.1186/1742-6405-9-4

**Published:** 2012-02-10

**Authors:** Alan R Lifson, Elizabeth M Krantz, Patricia L Grambsch, Grace E Macalino, Nancy F Crum-Cianflone, Anuradha Ganesan, Jason F Okulicz, Anne Eaton, John H Powers, Lynn E Eberly, Brian K Agan

**Affiliations:** 1 Division of Epidemiology and Community Health, University of Minnesota, Minneapolis, MN, USA; 2 Division of Biostatistics, University of Minnesota, Minneapolis, MN, USA; 3 Infectious Disease Clinical Research Program, Uniformed Services University of Health Sciences, Bethesda, MD, USA; 4 Infectious Disease, Naval Medical Center San Diego, San Diego, CA, USA; 5 Infectious Disease, National Naval Medical Center, Bethesda, MD, USA; 6 Infectious Disease Service, San Antonio Military Medical Center, San Antonio, TX, USA; 7 National Institute of Allergy and Infectious Diseases, National Institutes of Health, Bethesda, MD, USA

**Keywords:** Highly active antiretroviral therapy, mortality, CD4+ lymphocyte count

## Abstract

**Background:**

Although highly active antiretroviral therapy (HAART) has improved HIV survival, some patients receiving therapy are still dying. This analysis was conducted to identify factors associated with increased risk of post-HAART mortality.

**Methods:**

We evaluated baseline (prior to HAART initiation) clinical, demographic and laboratory factors (including CD4+ count and HIV RNA level) for associations with subsequent mortality in 1,600 patients who began HAART in a prospective observational cohort of HIV-infected U.S. military personnel.

**Results:**

Cumulative mortality was 5%, 10% and 18% at 4, 8 and 12 years post-HAART. Mortality was highest (6.23 deaths/100 person-years [PY]) in those with ≤ 50 CD4+ cells/mm^3 ^before HAART initiation, and became progressively lower as CD4+ counts increased (0.70/100 PY with ≥ 500 CD4+ cells/mm^3^). In multivariate analysis, factors significantly (p < 0.05) associated with post-HAART mortality included: increasing age among those ≥ 40 years (Hazard ratio [HR] = 1.32 per 5 year increase), clinical AIDS events before HAART (HR = 1.93), ≤ 50 CD4+ cells/mm^3 ^(vs. CD4+ ≥ 500, HR = 2.97), greater HIV RNA level (HR = 1.36 per one log_10 _increase), hepatitis C antibody or chronic hepatitis B (HR = 1.96), and HIV diagnosis before 1996 (HR = 2.44). Baseline CD4+ = 51-200 cells (HR = 1.74, p = 0.06), and hemoglobin < 12 gm/dL for women or < 13.5 for men (HR = 1.36, p = 0.07) were borderline significant.

**Conclusions:**

Although treatment has improved HIV survival, defining those at greatest risk for death after HAART initiation, including demographic, clinical and laboratory correlates of poorer prognoses, can help identify a subset of patients for whom more intensive monitoring, counseling, and care interventions may improve clinical outcomes and post-HAART survival.

## Introduction

Although highly active antiretroviral therapy (HAART) has significantly reduced mortality in HIV-infected patients [1-3], some patients receiving therapy are still dying. Guidelines for patients on stable HAART recommend laboratory monitoring and follow-up every 3-6 months [4]. However, more frequent and intensive monitoring and care may be indicated for those at greatest risk of post-HAART adverse clinical outcomes. Although CD4+ count is an important determinant of subsequent risk of death, other clinical and laboratory parameters at HAART start, as well as factors such as older age, may also affect post-HAART survival [5-10].

The U.S. Military HIV Natural History Study (NHS) is a prospective observational cohort of consenting HIV-infected military personnel [11]. Although HIV-positive status is an exclusion criterion for enlistment, active duty personnel undergo repeat HIV screening every 1-5 years, allowing for early diagnosis of infection; those found HIV-positive after enlistment receive free HIV specialty care, including HAART, at referral military medical centers. With NHS participants now followed for up to 14 years after HAART initiation, we examined clinical, laboratory and demographic factors at HAART initiation that were associated with subsequent mortality

## Methods

Study cohort: We included active duty members and retirees with: (1) documented HIV serostatus; (2) CD4+ count within six months before HAART; (3) HAART initiation after July 1995, and after or within one month before NHS enrollment. The governing central institutional review board approved this substudy; NHS participants provide written informed consent.

Variables for analysis: We evaluated death reports from participating centers through November 2010. A National Death Index (NDI) match was also conducted to capture deaths through 12/31/06 among participants lost to follow-up. Patients not known to have died who remained under follow-up had follow-up period censored at date of last clinic visit; those lost to follow-up had follow-up censored at 12/31/06 (corresponding to NDI search).

"Baseline" for this analysis was date of HAART initiation. Baseline CD4+ count and HIV RNA level were values closest to HAART initiation in the six months before HAART. Clinical AIDS events were those in the Centers for Disease Control definition (CD4+ criteria excluded) [12]. Anemia was defined as hemoglobin < 12 gm/dL for women and < 13.5 gm/dL for men within three years before HAART. Body mass index (BMI) calculations used height and weight within one year before or up to 30 days after HAART initiation.

For 80% of participants with documented last negative and first positive HIV test dates, estimated HIV SC date was calculated as the midpoint. For 20%, date of first positive but not last negative test was available; SC dates were imputed based upon median times between last negative and first positive date for other cohort members with comparable first HIV positive dates [13]. Chronic hepatitis B virus (HBV) infection was defined as ≥ 2 positive HBV surface antigen tests ≥ 6 months apart, and hepatitis C virus (HCV) infection as a positive HCV antibody test, both determined prior to or within 30 days of HAART initiation. We defined HAART based on regimens with ≥ 2 ART classes or certain combinations of ≥ 3 nucleoside/nucleotide reverse transcriptase inhibitors (NRTI); other (non-HAART) ART was essentially mono or dual therapy.

Statistical methods: Mortality rates following HAART initiation were calculated per 100 person-years (PY) with exact Poisson 95% confidence intervals (CI). We used Kaplan-Meier summaries for cumulative mortality rates post-HAART, and Cox proportional hazards models to estimate associations between baseline covariates and mortality. Based upon visual evaluation of age at HAART start and survival, linear splines were used to model effects of age separately for those < 40 and ≥ 40 years of age.

Variables significant (p < 0.05) in univariate analyses were included in the multivariate model. The proportional hazards assumption was assessed by graphical inspection of the log-negative-log of the survival distribution plotted by log of survival time for covariate categories.

## Results

Characteristics at HAART initiation: Table 1 summarizes characteristics of 1,600 persons in this analysis. Median time from estimated HIV SC to HAART initiation was 4.8 years; median baseline values were: CD4+ = 326 cells/mm^3^, HIV RNA = 4.5 log_10 _copies/ml, BMI = 25.5 kg/m^2^. Six percent were chronically infected with HBV and 5% HCV antibody positive; 0.5% had both. Initial HAART regimens were NRTI + protease inhibitor for 59%, NRTI + non-nucleoside reverse transcriptase inhibitor for 34%, and another regimen for 8%.

**Table 1:** Post-HAART Mortality Rate by Demographic, Clinical and Laboratory Parameters Prior to HAART Initiation, U.S. Military HIV Natural History Study

	Number and % of Participants	Number of Deaths	Deaths/100 PY (95% CI)
Gender			

Male	1532 (96%)	182	1.53 (1.32, 1.77)

Female	68 (4%)	8	1.37 (0.59, 2.70)

Age at HAART Initiation (years)			

< 29	395 (25%)	27	1.11 (0.73, 1.62)

29-33	371 (23%)	49	1.53 (1.13, 2.02)

34-39	454 (28%)	50	1.28 (0.95, 1.69)

≥ 40	380 (24%)	64	2.17 (1.67, 2.77)

Race			

Caucasian	712 (45%)	81	1.40 (1.12, 1.75)

African American	686 (43%)	93	1.70 (1.38, 2.09)

Other	202 (13%)	16	1.27 (0.72, 2.06)

BMI at HAART initiation (kg/m^2^)			

< 25	642 (40%)	87	1.70 (1.36, 2.10)

25 to < 30	584 (37%)	54	1.23 (0.92, 1.61)

≥ 30	210 (13%)	16	1.07 (0.61, 1.74)

Unknown	164 (10%)	33	2.23 (1.54, 3.13)

Clinical AIDS event prior to HAART			

No	1468 (92%)	138	1.20 (1.01, 1.42)

Yes	132 (8%)	52	5.23 (3.90, 6.85)

CD4+ count at HAART initiation (cells/mm^3^)			

≤ 50	112 (7%)	53	6.23 (4.67, 8.15)

51-200	259 (16%)	48	2.19 (1.61, 2.90)

201-349	517 (32%)	42	1.15 (0.83, 1.56)

350-499	433 (27%)	30	0.89 (0.60, 1.27)

≥ 500	279 (17%)	17	0.70 (0.41, 1.12)

HIV RNA at HAART initiation (copies/ml)			

< 1000	139 (9%)	10	0.80 (0.38, 1.47)

1000-9999	316 (20%)	23	0.86 (0.55, 1.29)

10,000-99,999	752 (47%)	79	1.38 (1.09, 1.72)

≥ 100,000	393 (25%)	78	2.75 (2.18, 3.44)

Anemia within 3 years			

No	941 (59%)	60	0.87 (0.66, 1.12)

Yes	635 (40%)	127	2.34 (1.95, 2.79)

Unknown	24 (2%)	3	1.96 (0.40, 5.73)

Chronic HBV or HCV antibody positive			

No	1385 (87%)	138	1.29 (1.09, 1.53)

Yes	166 (10%)	48	3.44 (2.54, 4.56)

Unknown	49 (3%)	4	0.95 (0.26, 2.42)

Year of HIV diagnosis			

Before 1996	848 (53%)	167	2.00 (1.71, 2.33)

1996 or after	752 (47%)	23	0.56 (0.35, 0.83)

Year of HAART initiation			

Before 2000	967 (60%)	171	1.74 (1.49, 2.02)

2000-2010	633 (40%)	19	0.72 (0.43, 1.13)

Time from SC to HAART initiation (years)			

< 2	473 (30%)	16	0.55 (0.31, 0.89)

2-5	353 (22%)	31	1.20 (0.82, 1.71)

5 to < 9	383 (24%)	64	1.83 (1.41, 2.33)

≥ 9	391 (24%)	79	2.26 (1.79, 2.82)

Other (non-HAART) ART			

No	835 (52%)	30	0.61 (0.41, 0.87)

Yes	765 (48%)	160	2.12 (1.80, 2.48)

**Figure 1:**
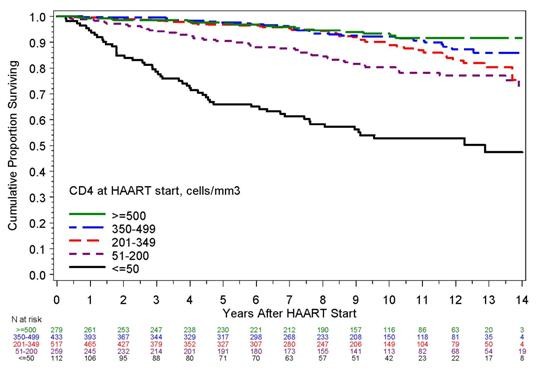
**Cumulative Proportion Surviving by Baseline CD4+ Count Prior to HAART Initiation, U.S. Military HIV Natural History Study**.

Mortality rates: Median follow-up post-HAART was 8.7 years, or 12,486 PY. During follow-up, there were 190 deaths, or 1.52 deaths per 100 PY (95% CI 1.31, 1.75). Cumulative mortality was 5% (95% CI 4%-6%) at 4 years, 10% (95% CI 9%-12%) at 8 years, and 18% (95% CI 15%-20%) at 12 years. Mortality was highest in those with ≤ 50 cells/mm^3 ^prior to HAART, and became progressively lower as CD4+ count increased (Table 1, Figure 1). Mortality rates progressively increased with greater baseline HIV RNA levels and longer time from HIV SC to HAART initiation. Mortality rates by other baseline factors are in Table 1.

Predictors of mortality: In univariate analysis, participants with CD4+ ≤ 50 cells/mm^3 ^and 51-200 cells/mm^3 ^at HAART initiation had significantly (p < 0.05) increased mortality compared to ≥ 500 CD4+ cells (Table 2). Mortality significantly increased with older age among those ≥ 40 years, but not among those < 40 years. Other factors significantly associated with increased mortality included clinical AIDS events before HAART, greater HIV RNA levels, anemia, HCV/chronic HBV, HIV diagnosis before 1996, HAART initiation before 2000, longer time from SC to HAART initiation, and non-HAART ART use (Table 2).

**Table 2:** Univariate and Multivariate Model Estimates for Post-HAART Mortality by Baseline Parameters Prior to HAART Initiation, U.S. Military HIV Natural History Study

Baseline Covariate (at HAART initiation)	Univariate Hazard Ratio (95% CI)	Multivariate Hazard Ratio (95% CI)
Gender: Female vs. Male	0.89 (0.44, 1.80)	--- †

Age:		

per 5 years older if age < 40 years	1.11 (0.95, 1.30)	0.91 (0.76, 1.08)

per 5 years older if age ≥ 40 years	1.27 (1.12, 1.44)**	1.32 (1.16, 1.51)**

Race		

Caucasian	1.00 (Reference)	--- †

African American	1.22 (0.91, 1.65)	--- †

Other	0.92 (0.54, 1.58)	--- †

BMI		

< 25	1.00 (Reference)	--- †

25 to < 30	0.73 (0.52, 1.03)	--- †

≥ 30	0.64 (0.38, 1.10)	--- †

Unknown	1.29 (0.87, 1.93)	--- †

Clinical AIDS event prior to HAART	4.26 (3.09, 5.87)**	1.93 (1.33, 2.81)**

CD4+ count at (cells/mm^3^)		

≤ 50	8.91 (5.15, 15.40)**	2.97 (1.56, 5.65)**

51-200	3.07 (1.76, 5.35) **	1.74 (0.97, 3.12)

201-349	1.69 (0.96, 2.98)	1.39 (0.79, 2.46)

350-499	1.29 (0.71, 2.35)	1.21 (0.66, 2.19)

≥ 500	1.00 (Reference)	1.00 (Reference)

HIV RNA (copies/ml) per one log_10 _increase	1.72 (1.44, 2.04)**	1.36 (1.12, 1.64)*

Anemia within 3 years		

No	1.00 (Reference)	1.00 (Reference)

Yes	2.61 (1.92, 3.55)**	1.36 (0.97, 1.92)

Unknown	2.29 (0.72, 7.30)	1.96 (0.40, 5.73)

Chronic HBV or HCV antibody positive		

No	1.00 (Reference)	1.00 (Reference)

Yes	2.61 (1.88, 3.63)**	1.96 (1.39, 2.76)**

Unknown	0.73 (0.27, 1.99)	0.98 (0.36, 2.67)

Year of HIV diagnosis		

Before 1996	3.35 (2.16, 5.21)**	2.44 (1.51, 3.93)**

1996 or after	1.00 (Reference)	1.00 (Reference)

Year of HAART initiation		

Before 2000	2.13 (1.31, 3.46)*	--- ††

2000-2010	1.00 (Reference)	--- ††

Time from SC to HAART (years) per 1 year increase	1.10 (1.06, 1.14)**	--- ††

Other (non-HAART) ART	3.28 (2.21, 4.87)**	--- ††

* p = 0.002 ** p < 0.001

† Multivariate model included only variables significant in univariate analysis

†† Not included in multivariate model because of strong collinearity with Year of HIV diagnosis

Exploratory analysis found strong colinearity between years from SC to HAART initiation, HIV diagnosis era, era of HAART initiation, and other ART use. For multivariate models, we chose HIV diagnosis before or after 1996, when HAART generally became available. Multivariate factors significantly associated with mortality included: increasing age among those ≥ 40 years, clinical AIDS event before HAART, CD4+ ≤ 50 cells/mm^3^, greater HIV RNA level, HCV/chronic HBV, and HIV diagnosis before 1996 (Table 2). Anemia within three years prior to HAART (p = 0.07), and CD4+ = 51-200 cells/mm^3 ^(p = 0.06) were of borderline significance.

In additional exploratory analyses, we added gender and race (as potential confounders), or initial HAART regimen class to our multivariate model; none of these was significantly associated with mortality.

## Discussion

Among HIV-infected military personnel, factors at HAART initiation associated with poorer survival included greater age in those ≥ 40 years, lower CD4+ count, greater HIV RNA level, prior clinical AIDS event, HIV diagnosis before 1996, and either HCV or chronic HBV; in multivariate analysis, anemia was borderline significant.

Consistent with other studies [14-16], mortality rates progressively decreased from lowest to highest baseline CD4+ strata, from 6.23 deaths in ≤ 50 CD4+ cell strata to 0.70 deaths/100 PY in the ≥ 500 CD4+ strata. While we did not find statistically significant mortality differences between higher CD4+ levels [8], this may reflect limited power from smaller numbers of deaths in higher CD4+ categories.

Higher HIV RNA level prior to HAART predicted increased mortality even after adjusting for CD4+ count and prior clinical AIDS. One potential explanation is if those with higher HIV RNA levels had longer time to viral suppression after HAART initiation, and/or did not develop viral suppression before death. Ongoing HIV replication may place patients at greater risk of opportunistic disease or death [17,18].

Among those ≥ 40 years, older age at HAART start was associated with poorer prognosis. Other analyses of HIV patients in the HAART era also report decreased survival with older age [8-10,18]. Given increasing recognition of HIV in those ≥ 50 years [19,20], older patients initiating HIV care and treatment require more intensive monitoring and follow-up.

In this analysis, we combined chronic HBV and HCV antibody positivity, since HIV patients co-infected with either virus have increased mortality from liver-related deaths [21-23]. Although we did not have access to HCV RNA, the great majority of HCV antibody-positive HIV patients likely had chronic HCV infection [23,24]. Providers should know current guidelines for co-infected patient including, when appropriate, therapy against HBV or HCV [4,23,25].

This cohort of HIV-infected military personnel has several strengths. Medical and other support provided to participants (including free HIV treatment) minimize socioeconomic disadvantage and lack of access to care that might otherwise influence outcome. Viral suppression rates in this cohort were reported to approach those in clinical trials [11]. The cohort is ethnically diverse. We can estimate time from HIV SC to HAART start. Follow-up extended for some patients to 14 years, longer than many other studies.

Our analysis has several limitations. We focused on baseline factors at HAART initiation, and not time-updated post-HAART changes. Our endpoint was overall mortality, rather than specific causes of death. Group differences in mortality may be due to additional unmeasured confounding factors. Populations with different demographic or risk characteristics may have different predictors of survival.

In summary, although HAART has improved survival for persons with HIV, defining those at greatest risk for death after HAART initiation, including demographic, clinical and laboratory indicators associated with poorer prognoses, can help identify a subset of patients for whom more intensive monitoring, counseling, and care interventions may improve clinical outcomes and post-HAART survival.

## Competing interests

The authors declare that they have no competing interests.

## Authors' contributions

AL was lead author on planning and coordinating the analysis, and drafting interim and final versions of the manuscript. EK, PG, AE, and LE provided statistical support and assistance with various aspects of the analysis. GM, NC, AG, JO, BA had multiple responsibilities related to this cohort, including data collection and oversight at study sites at which participants were followed. Co-authors all provided feedback and offered valuable comments and suggestions on versions of this manuscript. All co-authors have seen and approved the final version of this manuscript.
